# Cognition of relational discovery: why it matters for effective far transfer and effective education?

**DOI:** 10.3389/fpsyg.2023.957517

**Published:** 2023-04-28

**Authors:** Hilary J. Don, Micah B. Goldwater, Evan J. Livesey

**Affiliations:** ^1^School of Psychology, University of Sydney, Camperdown, NSW, Australia; ^2^Division of Psychological and Language Sciences, University College London, London, United Kingdom

**Keywords:** relational learning, far transfer, *complex discriminations*, category learning, individual differences

In his essay “*The Aims of Education*,” Alfred North Whitehead (1929) famously cautioned against “inert ideas.” He argued that an idea that was simply memorized and not used—in the sense of relating it to other ideas or novel situations as they arise—was potentially worse than learning no idea at all. The assumption at the core of this assertion is that understanding requires a continual process of assessing, comparing, relating, and extrapolating, and any “knowledge” that discourages this process is effectively harmful to the cognitive development of the individual and to their education specifically. The aim of education was, thus, to nurture this understanding and not to impart a series of seemingly disconnected facts that serve little purpose beyond their immediate sphere of relevance.

Nearly a century later, Whitehead's argument still resonates. Researchers in education, psychology, and cognitive neuroscience are still in search of a complete understanding of the processes that allow us to mentally represent abstract concepts such that they can be discovered, compared, and applied to new domains. Relational discovery—the process of coming to understand how entities interact—is a key aspect of this process because our understanding of a complex system necessarily involves learning the relations among its components. Hence, there is immense value in recognizing similarities in how things fit together that cannot be gained from simply memorizing the specific instances in which we encounter them. As Goldwater and Schalk (2016) have highlighted, research on relational category learning in cognitive psychology is strongly aligned with these broad educational goals. A relational category is defined in terms of the relations among its component entities rather than the precise characteristics of those entities. A relational category is one whose members share a common relational structure but not necessarily common features (Gentner and Kurtz, 2005). For instance, *a catalyst* does not classify molecules by any particular set of internal properties but by their role in facilitating chemical reactions.

In this article, we explain the general approach taken by cognitive psychologists to study and understand relational categorization and discuss several recent developments in methodology and theory. Laboratory studies investigating this question often take the form of a train-then-transfer task in which participants first learn about several instances, often requiring them to make judgments and predictions accompanied by corrective feedback, followed by transfer problems that are related to the training material in carefully controlled ways. This research provides laboratory models for the inert knowledge problem (Whitehead, 1929), aiming to isolate the cognitive processes that produce or overcome inert knowledge. We provide examples of this general methodology in the sections below and discuss their relevance for educational settings.

A consistent finding from research using these tasks is that learners struggle to generalize what they have learned from situations that differ in superficial ways and often apply relational knowledge incorrectly if the surface features encourage them to do so (Gick and Holyoak, 1980, 1983; Spencer and Weisberg, 1986; Holyoak and Koh, 1987; Ross, 1987; Novick, 1988; Bassok and Holyoak, 1989; Gentner et al., 1993; Reeves and Weisberg, 1994; Anolli et al., 2001; Perkins, 2009; Gonzalez and Wong, 2012; Trench and Minervino, 2015). Thus, it seems that there is often competition for the learner to acquire and use information that is feature-specific vs. relational. At the same time, relational categories typically evolve from an understanding of (and comparison between) specific concrete instances. As such, although they may be defined in terms of their relational properties, they often correlate with more concrete features, or at least connote specific perceptual properties to the individual learner. For instance, as a relational category, a *conduit* could be any process, agent, or object that facilitates the secure and easy passage of the substance that travels through it (whether that substance is ideas, data, water, electricity, etc.). However, if one asks an electrician to describe a conduit, they will likely have a much more feature-specific class of objects in mind, namely forms of non-conductive tubing that are used to protect the electrical wire. The same can arguably be said of the problems that STEM students encounter in formal education; while the concept is often intended to be abstract and widely applicable, it needs to be taught in the context of specific examples. Relational concepts are rarely (if ever) completely independent of the featural context in which they are encountered. Indeed, it is likely that the specific featural contexts in which a relational category is first encountered are beneficial for its inception (Snoddy and Kurtz, 2021). Early in the learning of a concept, associations with specific stimuli may facilitate initial understanding but also help the learner recall the appropriate set of relational concepts while they are still developing or consolidating relational knowledge. As Goldstone and Barsalou (1998) note, it is possible that our abstract relational ideas are never fully divorced from the concrete featural content in which those ideas are first developed. Nevertheless, an ideal outcome for later learning is to achieve not only rich context-specific knowledge for a domain of expertise but also a deep and abstract understanding that would allow the flexible application of knowledge to novel contexts and problems from other domains when needed. The goal was a coherent knowledge base at multiple levels of abstraction.

Although the learning of specific instances and structural relations may be synergistic in many circumstances, we suggest that two related problems together pose a considerable challenge for students wishing to extract and use general relational principles. First, when discovering new underlying principles, feature-based learning may compete with and prevent the discovery of new relations. Second, when new relations apply to previously encountered features or when previously learned relations need to be applied to new features, attending to the wrong qualities of a problem prevents appropriate generalization. Although the second of these problems is at the core of students' difficulty with far transfer tasks, we argue that it cannot be considered in isolation without also recognizing the influence of the first.

To summarize, relational discovery and transfer have long been considered key components of education but, we argue, usually come with a set of correlated concrete features, which people also learn. In some cases, it may be *necessary* to learn the associations between concrete features of a learning instance before a common set of relations can be discovered across instances. This raises important questions about the functional and mechanistic relationships between relational and featural learning. Does featural learning necessarily need to precede relational discovery? Under what circumstances does featural learning facilitate vs. impede relational discovery and relational transfer?

## Relational discovery in the cognitive laboratory

In the remainder of this article, we discuss promising lines of research that address these questions using laboratory models of relational transfer. We focus here on research using categorization and predictive learning, two highly related paradigms with similarities both in terms of the methodologies used and in the hypothesized psychological processes responsible. Both categorization and predictive learning usually involve supervised learning, where the learner must make a predictive judgment on each trial, on the basis of the information presented, and are then given corrective feedback. In categorization, this takes the form of presenting a particular exemplar and asking the learner to classify to which category it belongs (a *classification* procedure). In predictive learning, participants are presented with one or several cues and must predict which outcome will follow. In practice and process, these forms of learning are very similar and, indeed, they appear to have similar properties. Next, we briefly describe several ways in which relational discovery and transfer have been explored using these methods.

## Relational discovery in categorization

**Figure 1:**
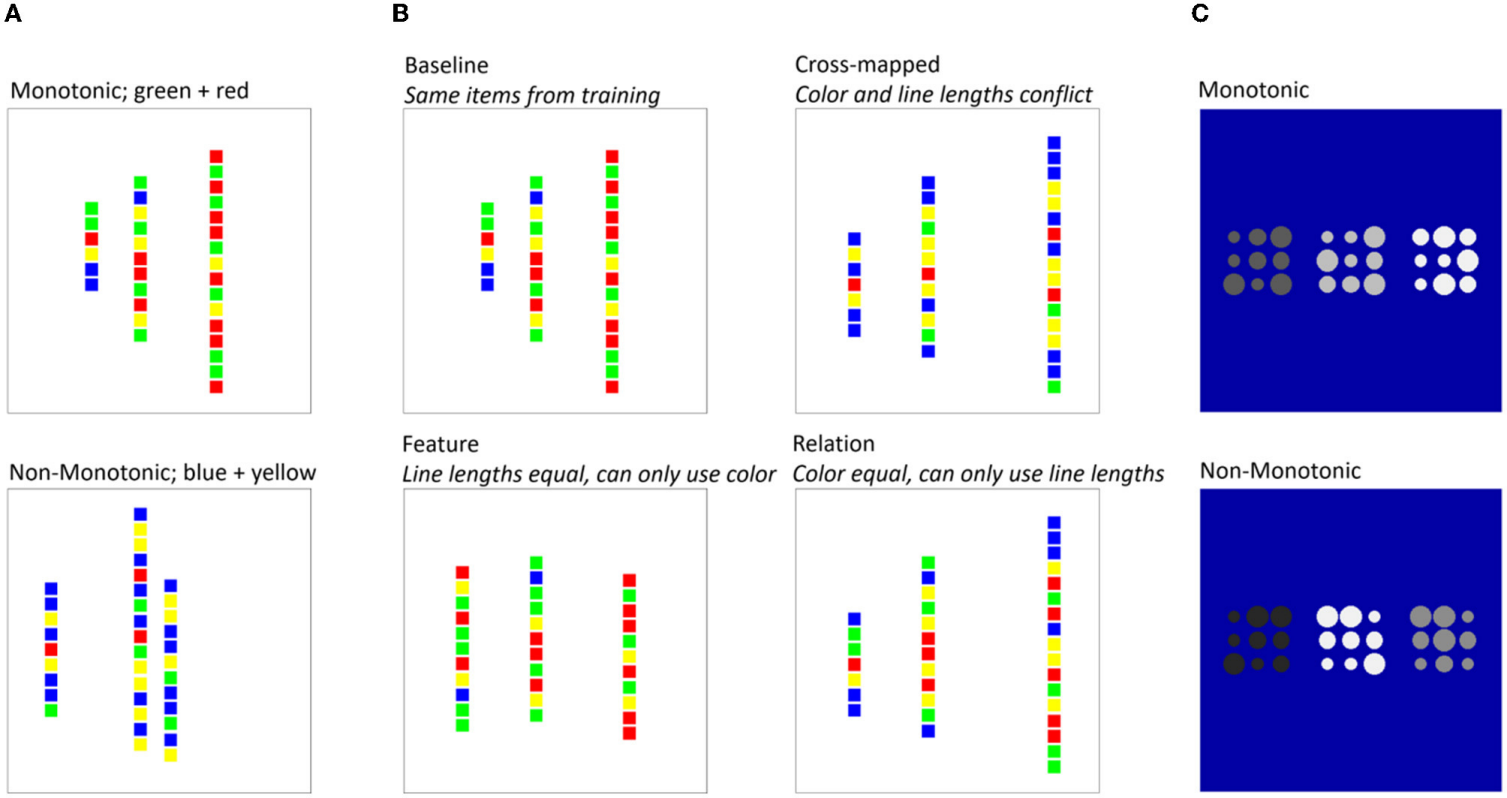
Example stimuli from a relational categorization experiment (Goldwater et al., 2018). **(A)** Participants are trained on exemplars that abide by a relational spatial rule (line lengths change monotonically for one category and non-monotonically for the other) but also have color proportions that correlate with category membership. **(B)** Participants are given “near” transfer test items that possess spatial relations and/or color proportions consistent with one or other category. **(C)** Participants are given a new “far transfer” categorization task in which a similar monotonicity rule applies, this time based on the shades of gray of the component features.

Relational categorization involves classifying an exemplar, usually comprising several components or events, in terms of the way its components relate to one another. Researchers have used visual classification tasks to allow relational categories to coincide (and/or compete) with rich visual characteristics of individual exemplars (e.g., Goldwater et al., 2018; Patterson and Kurtz, 2020). To use an example from our study, (Goldwater et al., 2018; see Figure 1) used artificial categories in which category membership was defined by a relational rule. Each exemplar comprised three lines of small colored squares, each of a different length; if the lines changed in length monotonically moving from left to right, then they belonged to one category, and if the lengths changed non-monotonically, then they belonged to another category. The surface features, in this case, the colors of the squares, also correlated with category membership and could be used to perform reasonably accurately on the task even without knowledge of the relational rule. The study aimed to determine which parameters of the task facilitated learning in the relational category and whether individual differences in learning strategy or cognitive ability would predict this facilitation. Most importantly, a far transfer test was included in which the relational monotonicity rule was applicable but on a completely different set of features; performance on this test is aided by knowing the relational rule, but knowledge of the correlation between precise features (i.e., colors of the squares) and category label is of next to no use.

The task included “near” transfer items, answered without corrective feedback, in which the relational and featural properties of the line exemplars were manipulated independently to gauge the degree to which individuals were using them. Across several experiments, Goldwater et al. showed that many individuals used the features to classify all near-test trials, while others consistently used the relations, but no participant succeeded in using both, even though doing so would be advantageous. This suggests competition for learning about the featural and relational category information; learning the feature information could effectively prevent a learner from acquiring the relational rule, and vice versa. Goldwater et al. also showed that verbal hints (Experiment 1) and manipulations of the salience of the features and relations (Experiment 3) had a significant impact on near-transfer performance in a predictable fashion. However, despite their obvious influence on how participants classified the line stimuli, these manipulations had negligible impact on far transfer. We will go on to describe some manipulations that *do* improve far transfer, but first, we introduce a complementary set of procedures that have emerged alongside categorization.

## Relational discovery in predictive learning

Predictive learning describes various tasks in which participants make explicit predictions about the occurrence of one or several outcomes based on cues presented at the beginning of each trial. For instance, in the food allergist task, participants are asked to predict whether or not a fictitious patient will suffer an allergic reaction when they have eaten the food presented in each trial. The learner's goal was to gain an understanding of how the cues are related to the outcomes so that they can make judgments about the cue and its ability to signal or generate the outcome. Although predictive learning shares many attributes in common with traditional category learning tasks, it has traditionally been tied more strongly to other theoretical traditions (e.g., associative learning). The relevance of predictive learning tasks to formal education might not seem immediately obvious; however, there are clear and important connections. For starters, predictive learning tasks form one of the most commonly used paradigms for studying causal cognition, which has clear relevance to STEM education. At the very least, predictive learning helps us to understand how children and adults engage in naïve investigations of the causal structure of their world, which forms the basis of the causal reasoning students bring to bear for the task of formal STEM education (for better or worse). The differences between naïve causal thinking and normative causal thinking in STEM offer challenges for educators but understanding this naïve causal thinking is critical for effective instructional design that ideally can bridge these gaps (Clement, 1993; Chi et al., 2012).

In this domain, attempts to separate relational from feature-based learning and transfer have made use of learning tasks in which the solution cannot be derived from a simple linear sum of the outcomes predicted by individual cues (these are often referred to simply as *complex discriminations*). For instance, negative patterning involves a situation in which cue A leads to an outcome, cue B leads to an outcome but cues A and B together lead to the omission of that outcome. This pattern goes against what would be expected by the default modes in which people and other animals tend to generalize from past instances. It is not so much that feature-based models of learning *cannot* explain the solution to such problems (there are several ways in which they can, see Whitlow and Wagner, 1972; Livesey et al., 2011; Thorwart et al., 2012, 2017) but rather that if a consistent pattern contradicting the usual trend is identified by the learner, then they may extract structural qualities of the task and extrapolate them to new learning instances.

**Figure 2:**
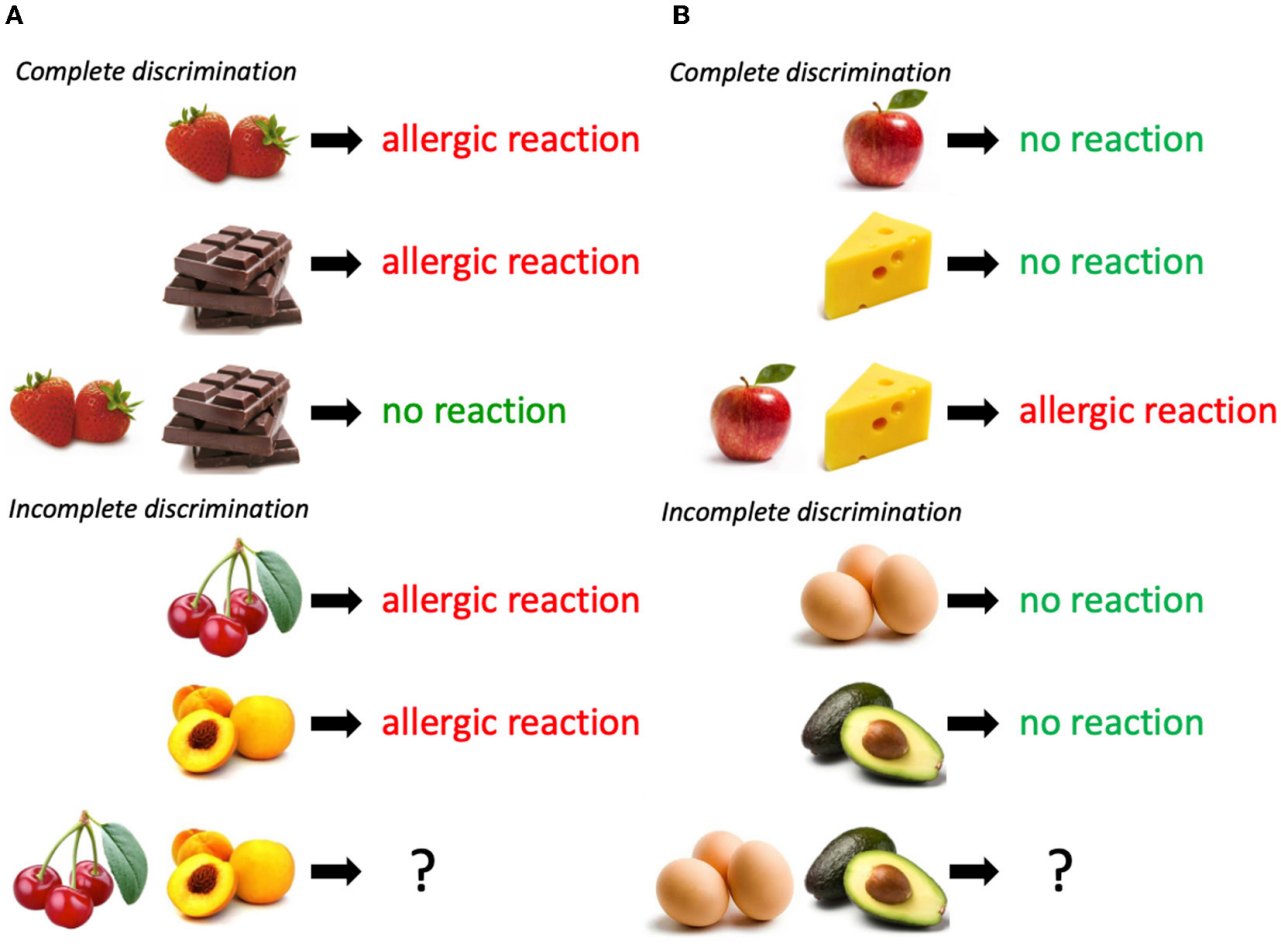
Schematic examples of some of the trial types used in the Shanks and Darby (1998) task. **(A)** depicts a negative patterning discrimination. **(B)** depicts a positive patterning discrimination. Each combination of food(s) and allergic reaction outcome constitutes one trial type that would be repeated several times across the course of a lengthy learning phase. Participants learn several “complete discrimination” examples of food-outcome contingencies that follow two variants (positive and negative) of a simple patterning rule. They also learn food-outcome contingencies which may or may not follow the same rule (incomplete discriminations). Participants are asked to use all the knowledge they have learned in the task to predict what will happen for the transfer items that involve combinations which they have not observed **(bottom row)**.

One prominent example of this type of approach was developed by Shanks and Darby (1998) (see Figure 2). Their task dissociates predictions of associative feature-based learning, on the one hand, and relational rule learning, on the other. Shanks and Darby used a food allergist task in which food cues eaten by a fictitious patient were sometimes followed by an allergic reaction outcome, sometimes not. The participant learns about many cue–outcome relationships, all of which follow a simple but counterintuitive set of rules: Two foods that result in allergic reactions when eaten on their own will not cause an allergic reaction when eaten together, whereas two foods that cause an allergic reaction when eaten together will not cause an allergic reaction when eaten on their own. This simple rule describes the relations between cues and outcomes in a way that makes the task of learning easier, overriding the feature-based generalization that participants typically rely on and, thus, avoiding confusion and interference (see Livesey et al., 2011; Thorwart and Livesey, 2016; Thorwart et al., 2017). Participants also learn an extra set of individual cues. On critical test trials, they are asked what will happen when these cues occur in combination. Learners using feature similarity to guide their judgments predict similar outcomes to those they have witnessed; for instance, if cue X leads to outcome 1 and cue Y leads to outcome 1, then cues X and Y together will also lead to outcome 1. However, learners who use the relational rule make the opposite prediction; if X leads to outcome 1 and Y leads to outcome 1, then X and Y together will lead to outcome 2. Our research (Don et al., 2016, 2020) has found that even though most participants can articulate the opposite rule post-experiment, only around half make generalized predictions based on the rule. Their tendency to do so is related to measures of cognitive reflection, strategic model-based choice in reward learning tasks, and working memory resources (Wills et al., 2011a,b; Don et al., 2015, 2016, 2020). There is also evidence that associative and relational predictions co-exist when participants are asked to make speeded vs. self-paced judgments (Cobos et al., 2017). A key feature of the Shanks–Darby task (and one that sets it apart from many of the categorization examples noted earlier) is that in order to learn the relational rule, the individual must also learn the basic predictive cue–outcome relationships well. This means that effective learners have a strong basis for conflicting predictions as they learn. This task is therefore useful for determining how predictions from these two processes generate different expectancies (measurable in brain and behavior) in the lead-up to making an explicit prediction.

Other studies have made use of similar complex learning tasks to explore how structural relations might be learned and transferred. (Livesey et al., 2019; Experiment 2) trained participants to make predictions in a similar task to that used by Don et al. (2020). On each trial, the learner always observed two foods and, on their basis, had to predict which of the two outcomes would follow. However, this time the relationship between food cues and reaction outcomes followed different sets of structural relations. For instance, for one group of food cues, there was always a predictive cue and an irrelevant cue within the pair that were shown. For another set of food cues, the correct answer could only be derived from the combination of foods that were shown (following biconditional discrimination, which has similar properties to the patterning example described earlier). In the second phase, the learner was presented with the same cues but re-arranged in new biconditional discriminations, with new outcomes. In this second phase, the cues that had been learned about as part of biconditional discrimination in the previous phase were initially learned about faster in the second phase, even though the concrete associations learned about in the first phase could not be generalized in a beneficial way to the second phase. This suggests that participants engaged in relational realignment as a consequence of learning the biconditional discrimination but did not immediately transfer it to all of the cues to which it applied. The results such as this potentially point to the manner in which relational categories emerge and generalize (initially in a concrete feature-based way), though more research is needed to ascertain how this process unfolds.

## What we can learn from relational discovery in categorization and predictive learning?

New results in these two domains are beginning to paint a complex picture of relational discovery the process of coming to understand how entities interact as a process that is both reliant on and in direct competition with the learning of concrete features of the task. We will describe some of these emerging patterns below. However, one might ask the more general question, why does the cognition of relational transfer matter for effective far transfer and effective education?

The same sorts of tendencies for feature dominance that we encounter in the laboratory are likely to be experienced by learners in education settings. Although the tasks employed in categorization and predictive learning contexts are artificial and usually rely on a rigid respond-and-correct sequential learning format, they still possess qualities that make them similar to classroom learning and assessment tasks. The dual goals of these tasks as they are presented to the learner—namely to understand some underlying set of relations and to make correct answers to a series of questions—are essentially the same as those that students face (albeit to varying degrees) in the classroom. Furthermore, the tendency to learn the concrete over the abstract, which is evident in many of these tasks, is displayed by healthy adults engaged in tertiary education. In Goldwater et al. (2018), Experiment 4 gave a questionnaire to the participants frequently used in education research concerning their study strategies (a subscale of the MSLQ; Pintrich et al., 1993). Participants who reported engaging in more elaborative study strategies, such as looking for connections between lecture and reading assignments (without any particular scaffolding by the teacher to do so), were more likely to learn the relations in comparison to participants who indicated they did not engage these strategies (who, in turn, were more likely to just learn the features). These participants were students at a selective and prestigious university, thus, it would be fair to assume that the autonomous use of elaborative strategies would be lower in other populations. What do these studies point to in terms of general principles that might aid in relational discovery in the classroom? Work on relational discovery in these domains reveals evidence of general abiding principles but also a clear need for further research. We will note several prominent examples here.

## Sequencing of information during learning

In categorization and predictive learning, as in most real-world settings, the information relevant to a key relational concept is rarely available all at once. Rather, it is distributed across multiple learning instances. The manner in which this information is distributed sequentially may have a strong impact on relational discovery. Recent results suggest that the “optimal” sequence for relational discovery does not necessarily match the conditions that are best for memorizing learning instances, rote learning essential information, or learning to discriminate between similar instances.

Interleaving schedules, in which there is a low chance of exemplars from the same category repeating across trials, are typically beneficial when tasks require discrimination between confusable categories (e.g., Bjork et al., 2013), as they promote between-category comparison (Rohrer and Pashler, 2010). Blocking schedules, in which there is a much higher chance of exemplars from the same category repeating across trials, are suggested to support learning the common relations across exemplars, as it promotes within-category comparison (e.g., Gentner, 2010). In our category learning study described earlier (Goldwater et al., 2018), we found that a blocking schedule improved relational discovery. A higher rate of alternation between exemplars of the monotonic and non-monotonic categories resulted in stronger learning of the superficial color features and their correlation with category membership whereas a higher rate of repetition of the same category was associated with the use of the relational rule.

Don et al. (2020) trained relatively simple (patterning) and relatively complex (biconditional) discriminations in interleaved, blocked, or clustered schedules. Clustering schedules involved presenting the constitutive elements of relational structures consecutively, which should assist the alignment of common relational structures between exemplars (e.g., Gick and Holyoak, 1983; Catrambone and Holyoak, 1989; Gentner et al., 2003; Goldwater and Gentner, 2015). Here, blocking was only beneficial for the transfer of the more complex biconditional discrimination, whereas clustering was beneficial for both types, regardless of individual differences in cognitive reflection. The difference in effects of trial sequencing could be explained by desirable difficulty (Bjork, 1994), that is, tasks that are more difficult can lead to better learning and retention (see Bjork et al., 2013), but these benefits disappear when the learning material is of sufficient complexity (e.g., Leahy et al., 2015). With simpler relational rules (such as patterning), blocking trials might be too simple for effective processing, while more difficult training sequences (such as clustering) could lead to better encoding and transfer of the relational rule. With a more complex relational rule (such as biconditional), more difficult training sequences may not be beneficial.

The evidence from these studies suggests that relations are easier to learn when the right amount of information is given in an uninterrupted sequence. However, what counts as the ideal amount of contiguous information (or grain size) may depend on the nature of the task and the underlying relation that needs to be learned. The sequencing considerations are a little different in the case of predictive learning than they are for categorization; in the case of predictive learning, it is often the way that predictive relations are experienced over different (but repeated) trial types that define the relational structure of interest. In contrast, in categorization, it is often the manner in which the simultaneously presented components of the exemplar are arranged that determines relational category membership. However, each trial provides a unique exemplar of this arrangement. In either case, comparison across trials is necessary to understand the abstract relations of interest and presenting relevant information in an uninterrupted sequence may be of importance for the discovery of those relations.

## Inference learning

As noted, most work in categorization and predictive learning uses a trial-and-error format in which the participant indicates what they think is the appropriate category or predicts which outcome will occur and then receives corrective feedback, a format we refer to here as classification. This classification format itself may promote a focus on perceptual features, at the expense of learning underlying relations. An alternative approach, sometimes referred to as inference learning, is to provide a partial category exemplar and information about the correct category and ask the learner to infer the missing components of the exemplar. A previous study on inference learning has shown that it benefits learning the statistical relations among exemplar features (e.g., Sakamoto and Love, 2010). Erickson et al. (2005) and see Higgins (2017) also showed that inference learning improved learning of abstract coherent categories compared to a typical classification approach. In our study (Goldwater et al., 2018), we found that inference learning promotes a focus on relations among stimulus properties and results in stronger relational discovery. In this study, we found that inference learning improved relational learning as well as far transfer but did not result in a more general performance advantage; participants in the inference learning conditions actually performed significantly worse at their learning task during training and on near transfer test were worse at classifying other exemplars that were similar to those seen during training. Thus, the advantage was specifically in identifying the underlying relational rule.

## Labeling and categorizing relational principles

The process of discovering and transferring abstract knowledge first requires learning appropriate relations that are represented as separate entities, independent of the features across which they are first encountered. Second, given a new isomorphic problem in which the same relational structure may be relevant, the learner must identify the appropriate way to map their relational knowledge to the new situation, potentially selecting among several candidate relations that could be relevant. Both the first and second parts of this problem present unique challenges. However, the strength and clarity of the relational representation are relevant factors in both cases. Kurtz and Honke (2020), Patterson and Kurtz (2020); and Snoddy and Kurtz (2021) have argued that far transfer requires the relational representation to reach category status, that is, the relation must be recognized as a known concept. What this affords for the learner is an efficient way to map specific instances of a given relation to a quickly identifiable category with known properties, rather than engaging in the potentially more effortful and error-prone process of generalizing from a series of less well-connected memories of previous (relationally) similar experiences. Similarly, our group's new work (in progress) shows that learning symbolic representations for relational categories facilitates transfer between disparate sets of features, compared to attempting to map the relations directly between feature sets (see, e.g., Gentner, 2010 for a thorough discussion).

## Progressive alignment

The analogical comparison provides a foundation for discovering, abstracting, and transferring structural relations, and a key component of this process is aligning the corresponding components of the two examples. Studies using humans and other animals have shown that providing several different instantiations of a relational concept increases the salience of abstract (e.g., same/different) properties of stimuli, and this effect increases when more unique instantiations are used (for a review, see Wasserman et al., 2017). In many cases, this might well be because presenting multiple instances results in a comparison process from which relational stimulus commonalities are either emphasized or discovered *de novo*. However, Gentner and Hoyos (2017) argue that what counts most for relational abstraction is not just any exposure to exemplars that share relational structure but having multiple *alignable* examples that conform to the same relational structure in a way that facilitates comparison between them.

There are multiple ways to make a pair of exemplars easier to compare. At least in young children, relational alignment is easier when there is a high featural similarity between the objects that require alignment. Evidence from a range of sources suggests that exposure to analogies where alignment is easier because the surface similarity of the components matches their relational similarities will facilitate more difficult structural alignment later (see Gentner et al., 1995; Kotovsky and Gentner, 1996; Gentner and Hoyos, 2017).

This method of scaffolding the analogical comparison process is referred to as *progressive alignment*. Notably, the facilitative effects of progressive alignment do not appear to rely on corrective feedback and may go unnoticed until far transfer is actually tested. There are several reasons why it may be effective. When the objects themselves are similar across two cases, this invites putting them in correspondence. This may help the learner then discover structural qualities that are only apparent when components are aligned appropriately. Alternatively, progressive alignment may shift our inductive biases toward looking for, and using, relational qualities, especially after initial alignments rely on the “training wheels” of more superficial similarity and attention has been shifted to more purely structural matches.

## Changing inductive biases to favor relational transfer

When faced with a novel (e.g., far transfer) task, the learner must decide which of many possible properties should form the foundation for transferring past knowledge. The same problem applies when identifying similarities between instances. For instance, Kroupin and Carey (2021) discuss this issue in the context of relational matching to sample, which entails identifying a relational attribute like “same” or “different” in the sample and choosing the option that contains the same relational attribute even if the surface features are very different. Kroupin and Carey note that people are *necessarily* heavily constrained by inductive biases, which allow them to select a subset of features that they consider likely to be relevant for matching (based on past experience). In at least some instances in which learners (e.g., crows, primates, and young human infants) fail to spontaneously produce relational same/different matching-to-sample behavior, it appears the same/different relation is simply not a feature that the learner is disposed to selecting even though they may have learned sufficient representations to support relational matching and, in principle, have the cognitive capacity to do so. This parallels the circumstances often faced by students encountering the inert knowledge problem. Although, in many cases, they have learned the appropriate relational concept and can apply it in some contexts, they simply fail to identify its relevance to a new situation.

Kroupin and Carey (2022) found that training on non-relational matching-to-sample tasks that still emphasize qualities that often form the foundations of relational comparison (e.g., size or number) could enhance relational matching to sample in adult humans. Since adult humans clearly hold (and spontaneously use) relations such as “same” and “different” in these tasks, this result can only be explained by changing inductive biases toward using some (e.g., same/different) features and inhibiting using others (e.g., color).

## Individual differences

When an educational intervention succeeds in improving outcomes, a key question that needs to be asked is who benefits the most? If the improvements are not universal, then they may level the playing field by improving the performance of weaker students or they may exacerbate differences between learners if they largely benefit more proficient learners. This is an important question in the domain of relational discovery specifically. Several psychological factors might predict how learners respond to a manipulation of the learning task. Here, we focus on two: first, *cognitive capacity*, broadly defined as proficiency in measures of fluid intelligence, working memory capacity, and analytical thinking, and second, *learning strategy*, which we conceptualize as the learner's natural disposition to search for underlying principles and rules as opposed to being content memorizing answers. These two factors are thought to be particularly relevant because concept learning is often considered the product of deeper or more involved engagement with the content. Where this assumption is accurate, it can be assumed that conceptual understanding will be a product of the learner's ability to engage at a deeper level, and their motivation to do so. Indeed, relational transfer in several contexts appears to be predicted by measures of cognitive capacity and learning strategy. Where a learning manipulation simultaneously improves relational transfer *and* removes the correlation between these personal variables and relational transfer, it suggests that the intervention has succeeded in elevating the performance of weaker learners in particular.

In relational categorization, several results suggest that learning strategy is a consistent predictor of relational discovery. McDaniel et al. (2014) found evidence that there are large and stable individual differences in individuals' tendencies to look for structure; participants who explicitly searched for rules in one learning task tended to learn underlying functions determining the answer in other content domains. Participants who were content to memorize examples to learn the correct answer tended to do so across multiple learning tasks. Little and McDaniel (2015a,b) found that this tendency to look for underlying rules benefitted learning regardless of whether the rule was feature-based or relational in nature, suggesting it is the strategy of searching for connecting principles that makes the difference rather than the precise content on which those principles are based. The tendency to search for rules did not appear to be related to fluid abilities (measured using Ravens Progressive Matrices) in Little and McDaniel's study. Goldwater et al. (2018) found a similar pattern of results in which rule searching strategy but not fluid ability predicted relational discovery across multiple versions of the classification task.

A key problem that is evident in recent results (e.g., Goldwater et al., 2018) is that although there are many ways to enhance the discovery of a relation, many seem to benefit those who seek out relations in the first place. In other words, one has to be generally disposed to look for underlying relations in order to benefit from the scaffolding that the experimenter (or instructor) provides. Goldwater et al. (2018) found that grouping the exemplars of the same category together improved performance on transfer tests, but this was mostly carried out by participants who reported that they were actively seeking rules in another learning task.

Are there methods that might benefit all, or even level the playing field by encouraging relational discovery, particularly in those less inclined to look for overarching rules? Some evidence suggests that inference learning might be particularly effective in encouraging relational discovery in those who do not naturally seek out rules. In our study of relational discovery using inference learning (Goldwater et al., 2018), we found that inference learning benefitted learners with self-reported suboptimal learning strategies (i.e., the students who did not engage in the elaborative learning strategies as mentioned above). Although this result needs further research, it suggests that the inference learning format assists relational learning for those who are not predisposed to engage in activities that promote relational learning generally. In contexts where learners' natural motivation to think more deeply about a problem may be low, or for classes where students seem satisfied with rote learning facts, inference learning may prove particularly valuable for encouraging conceptual understanding.

In contrast to the differences in learning strategy described above, relational transfer in some learning tasks seems to load much more heavily on fluid abilities and working memory. A consistent result found with the Shanks–Darby task, for instance, is that participants with higher working memory capacity, fluid ability as measured using Ravens, and cognitive reflection tend to use the relational rule much more readily on new transfer items. One thing that is different about this task compared to relational categorization examples is the need to hold information in working memory to even identify one *specific* instance of the structural relations governing the task, i.e., the learner needs to actively consider A+, B+, AB– (in contrast, the learner samples all the components that are related to one another all at once in the most relational categorization tasks).

## Future directions

The emerging patterns of results described above offer exciting avenues for future research but also provide hints at how a key aspect of effective education—the efficient transfer of relevant relational knowledge—might be improved for all learners. Key challenges remain for establishing how the collection of manipulations described above connects, especially the causal mechanisms by which they improve relational discovery and relational transfer.

### Prompting spontaneous relational discovery when motivation is lacking

One of the promising findings concerning inference learning is that it appears to elevate relational discovery in those who are not disposed to look for rules and or connecting principles. Studies have found little evidence that a rule-based strategy is correlated with ability *per se*, and it may be that learning strategy is associated more with motivational factors, such as the intrinsic value of satisfying a need for complex thought. If this is, in fact, the case, then it implies that the significant challenge is one of encouraging relational discoveries among those who are not motivated to search for such relations.

### Minimizing the difficulty of relational discovery and transfer by minimizing working memory load

In those instances where relational discovery *does* require a substantial investment of working memory or other cognitive resources, then the challenge is finding ways to minimize that load. The strategies that work best in this circumstance may be quite different from those that work when the main obstacle is motivation to search for rules.

### From competition to scaffolding

We have discussed the idea that ideal expert reasoning involves both rich context-specific knowledge (within their domain of expertise) and the flexible transfer of knowledge to novel contexts when needed. Tasks such as the one used in Goldwater et al. (2018) suggest that learning relations and learning features are in direct competition, and so the processes that support a more integrated knowledge base with multiple levels of abstraction are currently unclear. A study from Kemp et al. showed how learners can learn multiple levels of abstraction simultaneously, but the ease with which learners do so in these tasks suggests that they may be too simple to serve as laboratory models of real STEM learning (Kemp et al., 2010). Future studies will need to design appropriately challenging tasks where multiple levels of abstraction need to be learned and all levels of knowledge need to be maintained to support task performance (getting past the potential competition).

### From discriminating between categories to integrated systems of concepts

In typical laboratory category learning research, the task is to classify an exemplar as one of the small numbers of categories, where the relationship among these categories is simply how they differ from one another. The task is to learn to discriminate between the category options. However, in real knowledge domains, concepts and categories are related in meaningful ways beyond just how they differ. One could treat *catalysts* and *reagents* as contrasting roles in chemical reactions that chemistry students need to learn, but the schema (of catalytic chemical processes) that governs these roles is critical to understand, which entails understanding how these different relational categories relate to one another. Domain knowledge ideally is composed of an integrated system of relational concepts. Future research should try to reflect this desired goal state of expert knowledge.

### From cognitive laboratory to classroom

In order to be truly effective, any insights gained from studying relational discovery and transfer in the cognitive laboratory will need to have translatable benefits for the classroom. There is a cause for optimism in this regard. For instance, most of the manipulations discussed above are relatively easy and cost-effective to implement, and several have been shown to significantly improve understanding of real-world educational material. However, we acknowledge that translating insights in the laboratory to gains in the classroom is not trivial or straightforward. In the endeavor to learn materials that benefit all students—and particularly those who lack the motivation, strategies, or natural ability that could make spontaneous conceptual understanding easy—the cognitive study of relational discovery has a lot to offer. We argue that examining cognitive processes under carefully controlled conditions affords a unique level of understanding and one that can inform coordinated efforts to improve educational outcomes.

## Author contributions

HD, MG, and EL contributed to the conceptualization and writing of the article. All authors contributed to the article and approved the submitted version.
